# Proteomic Analysis of Gastrocnemius Muscle in Rats with Streptozotocin-Induced Diabetes and Chronically Exposed to Fluoride

**DOI:** 10.1371/journal.pone.0106646

**Published:** 2014-09-02

**Authors:** Aline Lima Leite, Janete Gualiume Vaz Madureira Lobo, Heloísa Aparecida Barbosa da Silva Pereira, Mileni Silva Fernandes, Tatiani Martini, Fernanda Zucki, Dóris Hissako Sumida, Alfredo Rigalli, Marília Afonso Rabelo Buzalaf

**Affiliations:** 1 Department of Biological Sciences, Bauru Dental School, University of São Paulo, Bauru, São Paulo, Brazil; 2 Department of Genetics and Evolution, Center of Biological Sciences and the Health, Federal University of São Carlos, São Carlos, São Paulo, Brazil; 3 Department of Basic Sciences, Araçatuba Dental School, UNESP – Universidade Estadual Paulista, Araçatuba, São Paulo, Brazil; 4 Bone Biology and Mineral Metabolism Laboratory, National Scientific and Technical Research Council (CONICET) School of Medicine, Rosario National University, Rosario, Santa Fe, Argentina; West Virginia University School of Medicine, United States of America

## Abstract

Administration of high doses of fluoride (F) can alter glucose homeostasis and lead to insulin resistance (IR). This study determined the profile of protein expression in the gastrocnemius muscle of rats with streptozotocin-induced diabetes that were chronically exposed to F. Male *Wistar* rats (60 days old) were randomly distributed into two groups of 18 animals. In one group, diabetes was induced through the administration of streptozotocin. Each group (D-diabetic and ND-non-diabetic) was further divided into 3 subgroups each of which was exposed to a different F concentration via drinking water (0 ppm, 10 ppm or 50 ppm F, as NaF). After 22 days of treatment, the gastrocnemius muscle was collected and submitted to proteomic analysis (2D-PAGE followed by LC-MS/MS). Protein functions were classified by the GO biological process (ClueGO v2.0.7+Clupedia v1.0.8) and protein-protein interaction networks were constructed (PSICQUIC, Cytoscape). Quantitative intensity analysis of the proteomic data revealed differential expression of 75 spots for ND0 *vs.* D0, 76 for ND10 *vs.*D10, 58 spots for ND50 *vs.* D50, 52 spots for D0 *vs.* D10 and 38 spots for D0 *vs.* D50. The GO annotations with the most significant terms in the comparisons of ND0 *vs.* D0, ND10 *vs.* D10, ND50 *vs*. D50, D0 *vs.* D10 and D0 *vs.* D50, were muscle contraction, carbohydrate catabolic processes, generation of precursor metabolites and energy, NAD metabolic processes and gluconeogenesis, respectively. Analysis of subnetworks revealed that, in all comparisons, proteins with fold changes interacted with GLUT4. GLUT4 interacting proteins, such as MDH and the stress proteins HSPB8 and GRP78, exhibited decreased expression when D animals were exposed to F. The presence of the two stress proteins indicates an increase in IR, which might worsen diabetes. Future studies should evaluate whether diabetic animals treated with F have increased IR, as well as which molecular mechanisms are involved.

## Introduction

The development of type 2 diabetes mellitus is critically related to insulin resistance (IR) [1]. IR is an impairment of insulin action in insulin-target tissues. It results from the inability of peripheral target tissues to respond appropriately to normal concentrations of circulating insulin and provokes impaired glucose tolerance despite elevated insulin concentrations [2], [3].

Fluoride (F) is a therapeutic agent that protects against dental caries [4]; therefore, it is added to public drinking water and dental products [5], [6]. However, studies in humans have shown that ingesting F in excessive doses can lead to glucose intolerance. The development of glucose intolerance depends on both the duration and dose of exposure to F [7], [8]. Oral ingestion of F leads to transient inhibition of insulin secretion in both rats and humans [9]. Impairment of glucose homeostasis occurs when plasma levels of F exceed 5 µM. In addition, plasma insulin levels increase as a function of the F concentration in drinking water [9]–[12]. Moreover, it has been shown that chronic F exposure can decrease the frequency pp185 tyrosine phosphorylation in muscle and white adipose tissues, while it increases the pp185 serine phosphorylation in white adipose tissue: these changes result in decreased insulin signaling [13], [14]. The negative effects of the chronic ingestion of F on glucose homeostasis can be ameliorated by physical activity [15]. Previous studies have reported that the retention of F is greater in animals with chemically induced diabetes [16]. However, the effects of chronic F administration on glucose homeostasis in animals with chemically induced diabetes have not been investigated. Fluoride has been shown to cause glucose intolerance [7], [8] and inhibit insulin secretion [9]; therefore, chronic F administration may exacerbate diabetes. If this is the case, then diabetic patients should be advised to reduce their F intake.

Skeletal muscle is the predominant tissue for insulin-stimulated glucose and lipid disposal, and it plays a crucial role in whole body IR. Defects glucose and lipid disposal are responsible for most of the IR observed in patients with type 2 diabetes [3], [17]. Proteomic analysis of skeletal muscle in exercise-trained, insulin-resistant mice has revealed alterations in the levels of abundance of proteins involved in molecular chaperoning, anti-oxidative stress response and mitochondrial functions [17]. Because F causes progressive degeneration of the structure and function of the skeletal muscles [18], it likely affects many proteins and enzymatic systems [19], [20]. However, the proteomic profile of skeletal muscle in animals with streptozotocin-induced diabetes and chronic exposure to F has not been investigated. We hypothesize that upon chronic exposure to F, rats with streptozotocin-induced diabetes will display significant alterations in the levels of skeletal muscle proteins involved in IR. Therefore, the aim of this study is to describe the global changes in the profile of protein abundance that occurs in rats with streptozotocin-induced diabetes and chronic exposure to varying concentrations of F introduced through the drinking water.

## Materials and Methods

### Animals and treatment

Male weanling *Wistar* rats (60 days old) were randomly distributed into two groups of 18 animals. In one group, diabetes was induced through the administration of streptozotocin (Sigma Aldrich, Saint Louis, MO, EUA) in a single intraperitoneal dose (50 mg/kg b.w., dissolved in saline, at 4°C) [21]. After 7 days, diabetes was confirmed by measuring blood glucose levels (Accu-Chek Performa, Roche Diagnostics, Mannheim, Germany). Each group, diabetic (D) and non-diabetic (ND), was further divided into 3 subgroups treated with different concentrations of F via the drinking water (0 ppm, 10 ppm or 50 ppm F, as NaF; designated as 0, 10 and 50, respectively). The animals were housed in groups of three per cage and received food and water *ad libitum* for 22 days. The temperature and humidity in the climate-controlled room, which had a 12 h light/dark cycle, were maintained at 23±1°C and 40%–80%, respectively. At the end of the study, the rats were anaesthetized with sodium thiopental (Thiopentax, Cristália Produtos Químicos e Farmacêuticos Ltda., Itapira, SP, Brazil). The gastrocnemius muscle was collected and stored at −80°C until proteomic analysis. All experimental protocols were approved by the Ethics Committee for Animal Experiments of Bauru Dental School, University of São Paulo (Protocol 13/2010).

### Sample Preparation for 2DE

The frozen tissue was homogenized in a cryogenic mill, model 6770 Freezer Mill (Spex, Metuchen, NJ, EUA). For the protein extraction, gastrocnemius muscle homogenate was incubated in lysis buffer (7 M urea, 2 M thiourea, 4% CHAPS, 1% IPG buffer pH 3–10, 40 mM DTT) supplemented with a protease inhibitor cocktail (Roche Diagnostics, Mannheim, Germany; 5 µL/mg of tissue) for 1 h at 4°C with occasional shaking. The homogenate was then centrifuged at 15,000 rpm for 30 min at 4°C and the supernatant containing the soluble proteins was recovered. The proteins were precipitated using the kit *PlusOne 2-D Clean-up kit* (GE Healthcare, Uppsala, Sweden), as recommended by the manufacturer. The resulting pellets were then resuspended in rehydration buffer (7 M urea, 2 M thiourea, 0.5% CHAPS, 0.5% IPG buffer pH 3–10, 18 mM DTT, 0.002% bromophenol blue). The protein concentration of each sample was measured by the Bradford protein assay [22]. After quantification, 1000 µg of muscle protein from each animal in a single test- group was pooled and submitted for proteomic analysis in triplicate, as described below.

### 2-D Separation

Skeletal muscle protein (1000 µg) was taken from each pooled sample and mixed with a rehydration buffer to a final volume of 400 µL. This sample was subsequently applied to immobilized, pH gradient strips (24 cm, pH 3–10) and focused in Ettan IPGphor 3 (GE Healthcare, Uppsala, Sweden) following the manufacturer's instructions. After focalization, the strips were equilibrated for 15 min with buffer (6 M urea, 75 mM Tris–HCl, 2% SDS, 29.3% glycerol) that was supplemented with 1% (w/v) DTT. The sample was then incubated for an additional 15 min with the same buffer supplemented with 2.5% (w/v) iodoacetamide (IAA). The second dimension analysis was performed in homemade, 12.5% acrylamide gels using the *Ettan DALTsix* (GE Healthcare, Uppsala, Sweden) electrophoresis system according to the manufacturer's recommended conditions. The electrophoresis was halted when the dye front reached the bottom of the gel. The resolved protein spots were then stained with Colloidal Coomassie Brilliant Blue G-250.

The gels were digitalized with an ImageScanner (GE Healthcare. Uppsala, Sweden), and all of the images were analyzed using the ImageMaster 2D Platinum 7.0 software (GE Healthcare, Uppsala, Sweden). Parameters used for automatic spot detection included: minimal area  =  4, smooth factor  =  2, and saliency  =  220. After detection, the spots were manually edited. The gel with the highest number of spots was chosen as the reference gel. The reference gel was then used to match the corresponding protein spots between other gels. The mean of normalized intensity values were run through the built-in ANOVA test in ImageMaster 2D Platinum 7.0 software (GE Healthcare, Uppsala, Sweden) to determine the significance of differential protein expression between the control and experimental groups. Spots that exhibited a statistical significance were excised from the gels.

### LC-MS/MS Analysis

After excision from the gel, spots were destained three times with 25 mM ammonium bicarbonate (Ambic)/acetonitrile (ACN) (50∶50 v/v) for 30 min. The destained gel pieces were dehydrated twice with ACN for 10 min and dried at room temperature (RT). The dried gel pieces were rehydrated with 20 mM DTT in 50 mM ambic for 40 min at 56°C. Excess reagent was removed and 55 mM IAA in a 50 mM ambic was added for 30 min at RT. Then, the remaining liquid was removed, and the gels were washed with 25 mM ambic, followed by dehydration with ACN. For digestion, the dried gels were incubated with 10 ng/µL trypsin in 25 mM ambic for 15 min (Trypsin Gold Mass Spectrometry, Promega, Madison, USA). The peptides were initially extracted from the gels in 50% ACN (v/v) with 5% formic acid, for 14 h at 37°C. The second extraction was performed in 50% ACN (v/v) with 1% formic acid for 15 min, followed by 60% methanol (v/v) with 1% formic acid for 15 min and rinsed twice with 100% ACN at 45°C under sonication (40 kHz/30 W, Branson, Danbury, USA). The extracts were dried using a vacuum concentrator (Eppendorf, Hamburg, Germany) and kept at −20°C. Prior to MS identification, dried peptides were dissolved in 10 µL of 0.1/3% formic acid/ACN. Peptides identification was performed on a nanoACQUITY UPLC-Xevo QTof MS system (Waters, Manchester, UK). The nanoACQUITY UPLC, was equipped with nanoACQUITY HSS T3, analytical reverse phase column (75 µm×150 mm, 1.8 µm particle size, Waters Manchester, UK) analytical reverse phase column. The column was equilibrated with mobile phase A (0.1% formic acid in water). Then, the peptides were separated with a linear gradient of 7–85% mobile phase B (0.1% formic acid in ACN) for 31 min at a flow rate of 0.4 µL/min. The column temperature was maintained at 35°C. The Xevo G2 Q-TOF mass spectrometer was operated in positive nanoelectrospray ion mode and data were collected using the MSE method in elevated energy (19–45 V), which allows data acquisition of both precursor and fragment ions, in one injection. Source conditions used included capillary voltage, 2.5 kV; sample cone, 30 V; extraction cone, 5.0 V and source temperature, 80°C. Data acquisition occurred over 20 min and the scan range was 50–2000 Da. The lockspray, used to ensure accuracy and reproducibility, was run with a [Glu1]fibrinopeptide solution (1 pmol/µL) at a flow rate of 1 µL/min, as a reference ion in positive mode at *m/z* 785.8427. ProteinLynx Global Server (PLGS) version 3.0 was used to process and search the continuum LC-MSE data. Proteins were identified with the embedded ion accounting algorithm in the software and a search of the *Rattus* database (reviewed only, UniProtKB/Swiss-Prot) downloaded on December 2013 from UniProtKB (http://www.uniprot.org/).

### Bioinformatics Analysis

Uniprot protein ID accession numbers were mapped back to their associated encoding Uniprot gene entries for each pair-wise comparison (ND0 *vs*. D0; ND10 *vs.* D10; ND50 *vs.* D50; D0 *vs*. D10; D0 *vs.* D50, Tables S1, S2, S3, S4 and S5, respectively, in File S1). Gene ontology annotation of broad biological process was performed using ClueGO v2.0.7 + Clupedia v1.0.8 [23], [24], a Cytoscape [25], [26] plugin. Briefly, Uniprot IDs were uploaded from Tables S1–S5 in File S1 and analyzed with the following default parameters: (1) enrichment (right-sided hypergeometric test) correction method using Bonferroni step down analysis mode, “Function”; 2) load a gene cluster list for *Rattus norvegicus;* (3), Evidence Codes set to “All”; (4) networking specificity set to “medium” (GO levels 3 to 8); and (5) Kappa Score Threshold set to 0.03. The protein-protein interaction network was downloaded from PSICQUIC [27], built in Cytoscape version 3.0.2, and constructed as proposed by Millan [26]. A network was constructed for each comparison described above (5 networks in total). These networks provide a global view of potentially relevant, interacting partners of proteins with changes in abundance.

### Statistical analysis

For proteomic data, statistical analysis was performed using *t* tests (p<0.05) available through the ImageMaster 2D Platinum 6.0 software (GE Healthcare, Uppsala, Sweden). Only proteins with significantly altered levels were excised for identification by MS.

## Results

### Identification of Differentially Expressed Proteins

**Figure 1:**
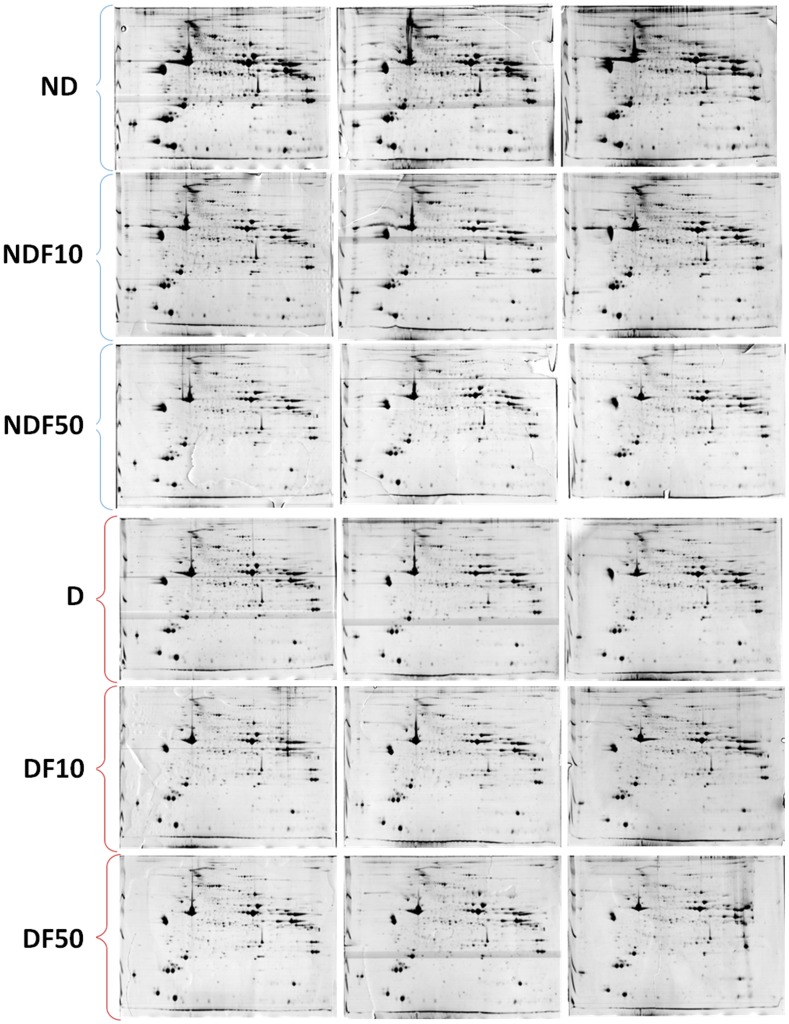
Profile of skeletal muscle proteins of diabetic rats treated with 0 ppm F (D0), 10 ppm F (D10) and 50 ppm F (D50), and of non-diabetic rats treated with 0 ppm F (ND0), 10 ppm (ND10) and 50 ppm F (ND50) in the drinking water for 22 days. IEF was performed using 24 cm immobilized linear pH 3–10 gradient strips, followed by SDS-PAGE with 12.5% polyacrylamide gels in the second dimension. Resolved proteins were visualized with Colloidal CBB G-250. Each 2-DE gel was performed from samples pooled from six animals for each group. The gels were done in triplicate.

Gels from skeletal muscle of the D0, ND0, D10, ND10, D50 and ND50 groups presented 415, 414, 414, 410, 412 and 415 total spots, respectively (Figure 1). The results of quantitative and qualitative intensity analyses are shown in Tables S1–S5 in File S1. In the comparisons between ND0 *vs.* D0, ND10 *vs.* D10, ND50 *vs.* D50, D0 *vs.* D10, D0 *vs.* D50, 75, 76, 58, 52 and 38 spots were differentially expressed, respectively, of which approximately 80% were successfully identified.

### Gene Ontology Annotation

**Figure 2:**
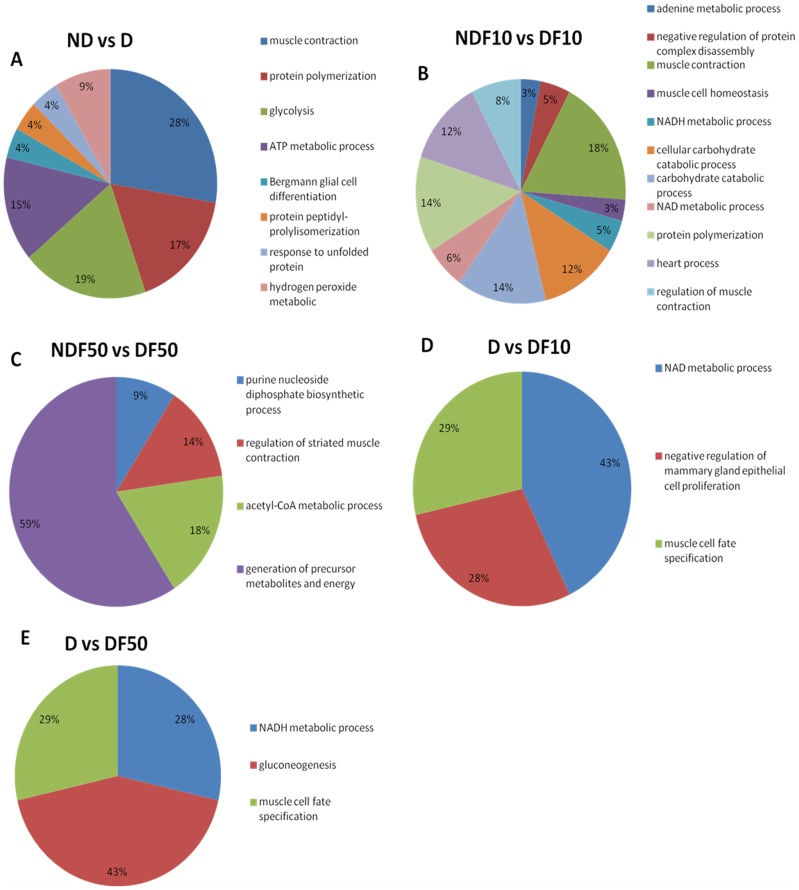
Functional distribution of proteins identified with differential expression in the skeletal muscle of diabetic and non-diabetic rats chronically treated with fluoride or not. Categories of proteins based on GO annotation Biological Process. Terms significant (Kappa = 0.03) and distribution according to percentage of number of genes association.

A functional classification, according to the biological process with most significant term, was determined for each comparison (Figure 2). The first 3 comparisons refer to diabetic (D) and non-diabetic (ND) animals exposed to water containing the same concentration of F in the drinking water. The comparison of ND0 *vs*. D0 (animals not exposed to F; Figure 2A) revealed 8 functional categories (glycolysis, muscle contraction, protein polymerization, ATP metabolic processes, response to unfolded proteins, Bergmann glial cell differentiation, protein peptidyl-prolylisomerization and hydrogen peroxide metabolic). Among these categories, muscle contraction (28%) had the highest percentage of associated genes. When the animals were exposed to 10 ppm F (ND10 *vs.* D10; Figure 2B), 11 categories were seen (NADH metabolic processes, NAD metabolic processes, heart processes, muscle cell homeostasis, regulation of muscle contraction, carbohydrate catabolic processes, protein polymerization, cellular carbohydrate catabolic processes, negative regulation of protein complex disassembly, muscle contraction and adenine metabolic processes). For the animals exposed to 50 ppm F (ND50 *vs.* D50; Figure 2C), 4 categories were identified (purine nucleoside diphosphate biosynthetic processes, acetyl-CoA metabolic processes, regulation of striated muscle contraction and generation of precursor metabolites and energy). The largest category, generation of precursor metabolites and energy, contained 59% of the associated genes.

The two final comparisons were between diabetic (D) animals exposed to F through the drinking water compared to their respective control (D, no F). When the D animals were exposed to 10 ppm F in the drinking water (D0 *vs.* D10; Figure 2D) only 3 categories were significant (NAD metabolic processes, negative regulation of mammary gland epithelial cell proliferation and muscle cell fate specification), with the largest category (43%) representing genes associated with NAD metabolic processes. Three categories were also observed for the group that was administered the highest dose of F (D0 *vs.* D50; Figure 2E) (muscle cell fate specification, NADH metabolic processed and gluconeogenesis), with the largest category (43%) associated with gluconeogenesis.

### Protein-Protein Interaction Network

**Figure 3:**
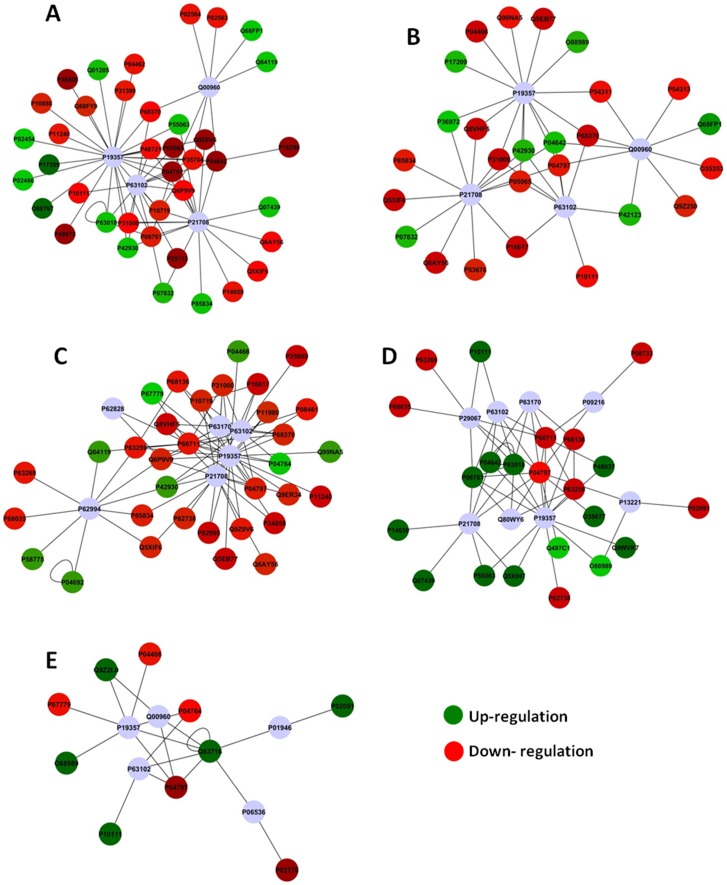
Subnetworks generated by JActiveModules for each comparison – A Group ND0 *vs.* D0; B Group ND10 *vs.* D10; C Group ND50 *vs.* D50; D group D0 *vs.* D10; E group D0 *vs.* D50. Red and green nodes indicate protein downregulation and upregulation, respectively, in the first group of each comparison.

For each of the comparisons discussed above, PSICQUIC was used to create one network, employing all of the interactions found,. After the global networks were created, nodes and edges were filtered using the specification for *Rattus norvegicus* taxonomy (10116). The p-values and the fold change values were added. The ActiveModules 1.8 plug-in for Cytoscape was used to generate Active Modules connecting subnetworks within the molecular interaction network generated from those genes presenting significant, coordinated differences in fold changes and p-value, as shown in the original proteomic analysis. The values for specific nodes and edges are shown in Table 1 for both the global network and the subnetworks. Figure 3 (A, B, C, D and E) shows the subnetworks generated by jActiveModules for each comparison. The majority of the proteins presenting with a fold change interact with GLUT4 (P19357; 7–26 proteins) and 14-3-3 protein zeta/delta (P63102; 4-14 proteins) for all analyzed comparisons. Proteins presenting fold change also interacted with Erk1 (P21708; 8–18 proteins) in 4 comparisons.

**Table 1:** Values of nodes/edges for each comparison considering the global network and the subnetworks (modules).

Groups	Global network	Module 01	Module 02	Module 03	Module 04	Module 05
ND0 *vs.* D0	739/1203	43/72	45/72	60/125	29/38	29/51
NDF10 *vs*. DF10	542/799	30/45	44/74	28/37	30/37	37/54
NDF50 *vs*. DF50	569/889	32/54	39/78	38/74	49/99	40/73
D0 *vs*. DF10	446/577	17/28	18/26	26/48	18/35	30/58
D0 *vs.* DF50	303/398	8/9	10/13	14/20	10/14	15/20

ND – non-diabetic rats; D – diabetic rats; 10 – 10 ppm fluoride in the drinking water; 50 – 50 ppm F in the drinking water.

## Discussion

To the best of our knowledge, this is the first study examining the effects of F provided through drinking water on rats with streptozotocin-induced diabetes. Previous studies have treated the animals with F for 6 weeks [4], [28]–[30]; however, a pilot study revealed high levels of mortality prior to completion of the experimental period. Therefore, an experimental time of 22 days was selected. The tested F concentrations, which were administered via drinking water, were designed to correspond to 1 ppm F (the concentration of F typically found in drinking water to prevent dental caries) and 5 ppm F (the concentration naturally present in the water of areas with endemic fluorosis). These concentrations had to be modified because rats metabolize F approximately ten times faster than humans [31]. The total number of protein spots identified in the muscle under the different conditions was similar to that observed for the same type of tissue in other studies [32].

Proteomic analyses reveal that the largest differences in expression, between ND *vs.* D rats with no exposure to F (ND0 *vs.* D0), occurred in genes related to muscle contraction. D rats displayed an increased expression of many types of myosin proteins (Mybpc1, Myh8, Myh6, Myh7, Myl3 and Mybph) (Table 2). The muscles of diabetic animals regularly need repair, which requires a higher concentration of contractile proteins [33]. Conversely, the expression of gelsolin, which is responsible for the regulation of actin cytoskeleton signaling was decreased in D animals (Table 2). Downregulation of gelsolin suggests a disruption in filamentous actin, which leads to the impairment of GLUT4 vesicle trafficking and glucose transport [32], [34].

**Table 2:** Proteins with expression significantly altered in gastrocnemium muscle of diabetic rats chronically treated with different doses of fluoride in the drinking water for 22 days.

*^a^*Access Number	Difference in expression (% volume)					Protein	Score	*^b^*pI/MW Theor.
	ND *vs*. D	NDF10 *vs.* DF10	NDF50 *vs.* DF50	D *vs*. DF10	D *vs.* DF50			
**P04462**	−1.385	___	___	___	___	Myosin-8 (Fragment)	393.3	29843.65/6.43
**P02564**	−1.385	___	___	___	___	Myosin-7	379.2	223083.03/5.64
**P02563**	−1.385	___	___	___	___	Myosin-6	358.4	223508.35/5.59
**P12847**	−1.385	___	___	___	___	Myosin-3	268.9	223857.70/5.64
**Q63518**	−2.369	−1.308	1.727	___	−1.115	Myosin-binding protein C_ slow-type (Fragment)	366.8	68736.75/6.41
**Q68FP1**	1.350	1.611	___	___	___	Gelsolin	100.7	86067.60/5.75
**O88599**	−1.088	___	___	___	___	Myosin-binding protein H	594.2	52656.34/5.99
**P42123**		1.158	___	___	___	L-lactate dehydrogenase B chain	224.1	36612.37/5.70
**P04642**	−1.776	___	___	1.394	___	L-lactate dehydrogenase A chain	853.1	36450.50/8.45
**P19629**	−1.776	___	___	___	___	L-lactate dehydrogenase C chain	221.8	35686.55/7.56
**P15429**	___	___	1.039	___	−1.258	Beta-enolase	1730.5	47013.90/7.08
**P04764**			1.039		−1.258	Alpha-enolase	1630.9	47127.86/6.16
**P07323**	___	___	1.039	___	−1.258	Gamma-enolase	1475.8	47140.53/5.03
**P11980**	___	___	−1.103	___	___	Pyruvate kinase isozymes M1/M2	1235.7	57817.79/6.63
**O35077**	*___*	*___*	*___*	1.474	*___*	Glycerol-3-phosphate dehydrogenase [NAD(+)]_ cytoplasmic	1686.7	37452.52/6.16
**O88989**	___	___	___	1.131	___	Malate dehydrogenase_ cytoplasmic	132.8	36483.11/6.16
**P04797**	−2.011	___	___	___	−1.358	Glyceraldehyde-3-phosphate dehydrogenase	70.8	35827.99/8.14
**P63018**	___	___	___	1.238	___	Heat shock cognate 71 kDa protein	2222.8	70871.07/5.37
**P06761**	___	___	___	1.238	__	78 kDa glucose-regulated protein	107.1	72346.99/5.07

Relative differential expression is indicated by no sign, when the protein is up-regulated and by - sign, when the protein is down-regulated in the respective comparison. ^a^Identification is based on protein ID from UniProt protein database (http://www.uniprot.org/). ^b^Theoretical pI/molecular weight (kDa) of theoretical protein. ND – non-diabetic rats; D – diabetic rats; 10 – 10 ppm fluoride in the drinking water; 50 – 50 ppm F in the drinking water.

The expression of genes related to catabolic processing of carbohydrates was most strongly altered when ND and D rats were exposed to 10 ppm F (ND10 *vs.* D10). The expression of L-lactate dehydrogenase B chain (Table 2) was increased in ND rats compared to D rats. Interestingly, in the absence of F, the expression levels of L-lactate dehydrogenase A chain and L-lactate dehydrogenase C chain were lower in ND rats than in D rats. A previous study reported the absence of L-lactate dehydrogenase A chain in the liver of rats (ND) treated with F [28]. Decreased expression of this enzyme leads to increased aerobic glycolysis. These data are consistent with the expectation that in the presence of a low F concentration, the levels of aerobic glycolysis should be lower in the muscles of D rats than in the muscles of ND rats.

When ND and D animals were exposed to 50 ppm F (ND50 *vs.* D50), expression changes were apparent in proteins related to generation of precursor metabolites and energy, including the enolases (Eno3, Eno1 (α) and Eno2), (Table 2). The data from this study reveal that the expression of enolases is downregulated in D animals. In a previous study, α-enolase expression was increased in the bladder smooth muscle of rats 2 months after the induction of diabetes mellitus with streptozotocin, although no change in expression was observed 1 week after treatment [35]. This increased expression observed 2 months after the induction of diabetes mellitus was hypothesized to be a protective mechanism to counterbalance hypoxia in long-standing cases of diabetes mellitus [36]. In contrast, results from the present study were obtained from muscle samples collected 22 days after diabetes induction, rather than 2 months. In addition, F has been shown to inhibit enolase activity by interacting with magnesium, a co-factor for this enzyme [37]. Therefore, the reduced expression of enolase in the D animals of the present study might result from the combined impacts of diabetes and exposure to F. F has also been shown to increase the expression of aldolase (an upstream glycolytic enzyme) in liver, which can be an attempt to increase the efficiency of glycolysis [28]. In the present study, the expression of pyruvate kinase isozymes M1/M2 (Table 2) (downstream to enolase) was higher in D rats than in ND. These results are consistent with those previously reported in the muscle of rats with type 2 diabetes [33]. In addition, there was increased expression of other enzymes involved in glycolytic pathway, such as pyruvate dehydrogenase E1 component subunit beta, mitochondrial and isoform R-type of Pyruvate kinase isozymes R/L. The increased expression of these enzymes could counterbalance the reduced expression of enolase.

When D animals were exposed to 10 ppm F (D0 *vs.* D10), the most noticeable changes in expression were observed in genes associated with NAD metabolic processes. The expression levels of glycerol-3-phosphate dehydrogenase [NAD(+)]_ cytoplasmic (GPD1) and Malate dehydrogenase_cytoplasmic (MDH) (Table 2) were lower in animals treated with F than in the controls. Previous studies have shown that F inhibits the activity of enzymes involved in the citric acid cycle [19]. In the liver of rats treated with 50 ppm F, MDH was not detected, which may contribute to reduced citric acid cycle flux [28], as well as the reduced expression of GPD1, which is a NAD(H)-dependent cytosolic enzyme. GPD1 catalyzes the conversion of dihydroxyacetone phosphate, derived from glucose, to glycerol-3-phosphate, which is finally acylated to form triglycerides [38]. Reduced expression of NAD-associated enzymes may contribute to the decreased consumption of NADH by the respiratory chain induced by F [39]. Fluoride reduces the consumption of oxygen and NADH in complex I of the respiratory chain. Therefore, higher concentrations of NADH could downregulate the enzymes that produce this reduced coenzyme. It has been shown that NADH downregulates MDH [40].

The D rats exposed to 50 ppm F (D0 *vs.* D50) displayed major changes in the expression of proteins involved in gluconeogenesis. Among these proteins, g lyceraldehyde-3-phosphate dehydrogenase (GAPDH), (Table 2) was upregulated following exposure to F. Increased expression of GAPDH is usually observed in diabetes [41], which is consistent with our findings of increased GAPDH expression in D animals compared o ND animals with no exposure to F. This increase intensified upon exposure to 50 ppm F, which may indicate the loss of glucose and lipid homeostasis. Insulin acts to decrease gluconeogenic enzymes. The increase in gluconeogenic enzymes in rats exposed to 50 ppm F may result from the effect of F on the insulin receptor, because F exposure has been shown to decrease the pp185 tyrosine phosphorylation status in muscle tissue [13]. In a study of the skeletal muscles of obese/overweight and morbidly obese women, GAPDH expression was increased. The authors hypothesized that this increase in GAPDH may compensate for the progressive decrease in the function of muscle mitochondria observed in obese individuals. Furthermore, higher levels of GAPDH may also contribute to the loss of glucose and lipid homeostasis and to the eventual development of type 2 diabetes [42]. Many studies have indicated that the administration of high doses of F may provoke changes in glucose homeostasis and lead to IR [9]–[11], [13], [14].

The analysis of subnetworks revealed that many proteins (Table 2) with fold changes interacted with GLUT4 (P19357) (figure 3), a protein involved in glucose uptake and trafficking. In a study by Scherp et al. (2012), 18 proteins interacting with GLUT4 were identified in human skeletal muscle culture following treatment with bioactives from *Artemisia dracunculus* L. Among these GLUT4-interacting proteins, 13 were up-regulated following insulin stimulation in the presence of bioactives [32]. In the present study, we found 26 altered proteins in our networks that interact with GLUT4 (Figure 3). The main goal of this study was to investigate the changes in expression that occur when animals with streptozotocin-induced diabetes are exposed to F; therefore, our discussion will focus on the subnetworks generated from the data of the comparison between D animals in the presence of F with D animals in the absence of F or not (Fig 3D and 3E). In the presence of F, many proteins with decreased expression, such as MDH, interact with GLUT4. Decreased function of these GLUT-4-interacting proteins may impair mitochondrial metabolism [43], thus altering the ability to switch between carbohydrates and fats as a source of oxidative energy. Furthermore, changes in the function of GLUT4-interacting proteins may alter how sensors of energy stress signal imbalance. One such sensor, AMP-activated protein kinase (AMPK) [44], signals via the transcriptional co-activator of peroxisome proliferator activated receptor, PGC-1α [45]. The latter induces mitochondrial biogenesis and increases the concentration of GLUT4 in the muscle [46]. However, F can cause changes in the expression of mitochondrial proteins [28], [29], leading to functional alterations in mitochondria [19], and altered mitochondrial function can lead to oxidative stress.

In the present study, stress-related proteins and anti-oxidant markers that interact with GLUT4 were down-regulated in D animals exposed to 10 ppm F. Downregulation of proteins, such as heat shock protein HSPB8 (P63018) and GRP78 (P06761), indicates a reduced tolerance to stress in D rats exposed to F. Oxidative stress also decreases the gene and protein expression levels of GLUT4. Decreased GLUT4 expression might be expected to result in a decrease of the glucose uptake, thus leading to IR [47]. When insulin-resistant mice are exposed to aerobic exercise, the expression of heat shock proteins increases. Therefore, exercise may play a key role in improving IR [17]. Similarly, when exposed to F, D animals display an increase in oxidative stress due to the concomitant reduction of mitochondrial proteins, such as MDH and the stress proteins HSPB8 and GRP78. The reduction of these two latter proteins indicates an increase in IR, which might exacerbate diabetes.

When the concentration of F is increased to 50 ppm, the stress proteins were not altered in D animals. Absence of a dose-dependent response was also reported in a study, where treatment of rats with 25 ppm F led to histological alterations in liver, while treatments with 5 or 50 ppm F did not [48]. This observation may be due to the organism adapting to a dose of F that, in the short term, might lead to high levels of toxicity but diminishes in toxicity over the long term [49]. In the future, the possibility of adaptation should be investigated at the molecular level. Here are some important limitations to our findings. First, the animal model of streptozotocin-induced diabetes differs from type-1 diabetes in humans. Second, this is the first study to evaluate the effects of chronic F exposure in animals with streptozotocin-induced diabetes. We observed alterations in muscle proteins related to glucose homeostasis in D animals treated with F. However, additional studies should evaluate whether D animals treated with F have increased IR and which molecular mechanisms are involved in IR before clinical recommendations can be made regarding diabetic patients.

## Supporting Information

File S1 **Supplementary information Tables S1 to S5.**
(XLSX)
